# Case report: Successful treatment of advanced colon cancer in an eighty-year-old man with long-term and multi-stage endoscopic minimally invasive therapy

**DOI:** 10.3389/fonc.2024.1367173

**Published:** 2024-02-20

**Authors:** Nana Zhang, Lulu Zhu, Yan Liu, Xiaolong Chen, Bifang Zhang, Chunhong Wen, Huayu Zhang, Qinglin Tang, Mingqing Zhang

**Affiliations:** Department of Gastroenterology, The 909th Hospital, School of Medicine, Xiamen University, Fujian, China

**Keywords:** colonic neoplasms, aged, endoscopy, argon plasma coagulation, surgery

## Abstract

**Background:**

No previous studies have reported on the use of minimally invasive endoscopic therapy for colon cancer in older patients.

**Case presentation:**

An 80-year-old man was admitted to our hospital with haematochezia and diagnosed with advanced colon cancer in 2018. Traditional surgical care was rejected by his family. We successfully treated the patient with multiple minimally invasive endoscopic therapies, such as argon plasma coagulation, from 2018 to 2021.

**Conclusion:**

Invasive endoscopic therapy is a feasible way to treat colon cancer in older patients.

## Introduction

Colorectal cancer (CRC) is the third most common cause of cancer mortality worldwide and the third leading cause of death in men aged over 80 years (1, 2). Older patients with CRC are often in an advanced stage at the time of diagnosis, losing the opportunity to undergo surgical treatment and substantially limiting their life expectancy. Moreover, many older patients have major comorbidities that may minimize or even negate the benefits of adjuvant chemotherapy or radiotherapy (3, 4). Choosing an appropriate method for treating CRC in older patients is a current challenge in clinical practice. This case report describes the successful treatment of an 80-year-old patient with advanced colon cancer using multi-stage endoscopic minimally invasive therapy with the intention of providing new ideas for the treatment of colon cancer in older patients.

## Case presentation

**Figure 1:**
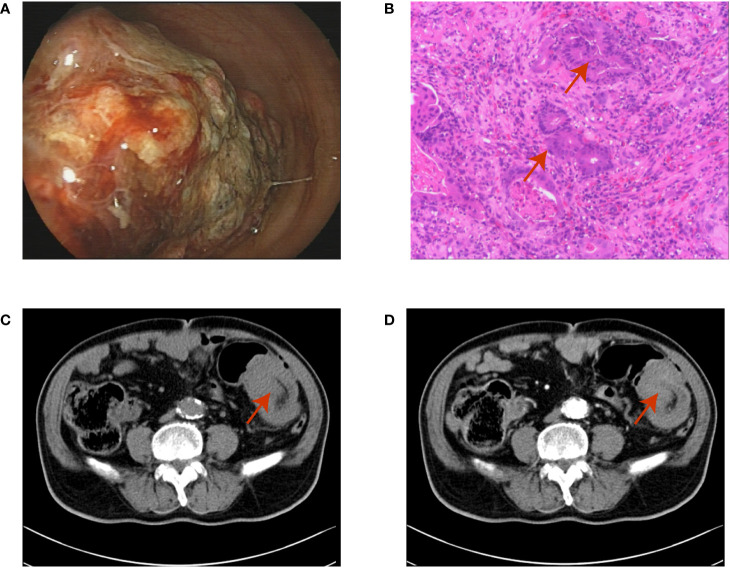
**(A)** The colonoscopy shows a mass blocked the lumen of the descending colon and the endoscope could not pass; **(B)** The haematoxylin-eosin staining of the biopsy shows adenocarcinoma (red arrow); **(C, D)** The contrast-enhanced abdominal computed tomography shows the colon wall thickening and the maximum cross-section of the mass is 4.4×7.0 mm (red arrow).

**Figure 2:**
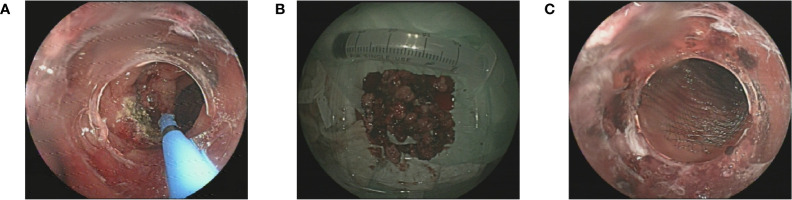
**(A)** The endoscopic resection and argon plasma coagulation of the tumour; **(B)** The excised tumour tissue; **(C)** The stent placed in the lumen.

**Figure 3:**
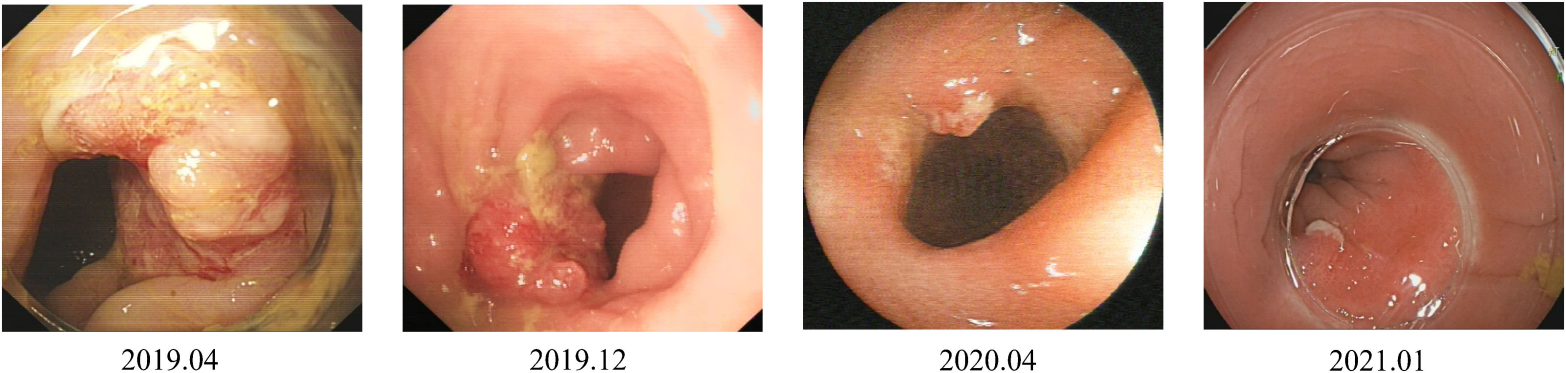
The size of the tumour in the colon has gradually shrunk over three years from 2019 to 2021 after endoscopic therapy.

**Figure 4:**
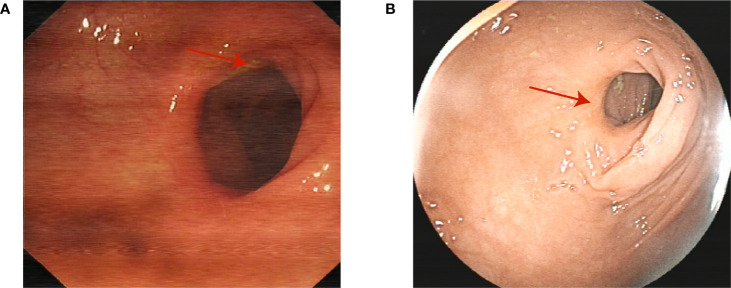
Scars are seen at the original tumour site in three **(A)** and four **(B)** years after the original diagnosis in 2018. (red arrow).

An 80-year-old Asian man with haematochezia for 10 days was admitted to our hospital in Nov. 2018 and diagnosed with advanced colon cancer, protruded type, clinical stage T2N0M0 (**Figure 1**). Colonoscopy revealed a mass in the descending colon that blocked the lumen, and blocked passage of the endoscope (**Figure 1A**). The biopsy specimen was friable and bled easily. Histopathological examination (H&E staining) showed deeply stained irregular nuclei with necrotizing tumour cells and revealed adenocarcinoma (**Figure 1B**). Contrast-enhanced abdominal computed tomography showed a thickening confined to the descending colon wall and no metastasis to the lymph nodes or abdominal organs (**Figures 1C, D**). The man had hypertension and coronary artery disease for more than ten years, type 2 diabetes mellitus for more than six years, and had been on long-term medication (unspecified) for years. He did not have a history of surgery or radiation exposure, and his family history was unremarkable. Considering the age and health status of the patient, his family rejected traditional surgery and neoadjuvant therapy because of potential complications like wound infection and kidney injuries, leading to the shortage of lifespan. Therefore, we aimed to prevent luminal obstruction and maintain the patient’s quality of life by using a disposable polyp snare, endoscopic resection, and argon plasma coagulation (APC) to remove part of the tumour (**Figures 2A, B**) and then placing a stent using the colonoscope (**Figure 2C**). From April 2019 to February 2021, the patient underwent a colonoscopy six times for the tumour in our hospital, including polyp resection, electro-coagulation and electro-section, and APC (**Supplementary Figure 1**). The tumour size in the colon gradually decreased and no other complications developed (**Figure 3**). During the entire process, the patient did not receive any other therapy, including chemotherapy, radiotherapy, or traditional Chinese medicine. Surprisingly, a regular examination with colonoscopy in July 2021 showed that only postoperative scars were seen at the original tumour site, and no tumour proliferation was found (**Figure 4A**). One year later, in May 2022, the patient underwent colonoscopy and still showed no colon cancer recurrence (**Figure 4B**). And the follow-up examination by abdominal ultrasounds and chest X-ray also showed no metastases or other diseases from 2019 to 2022. However, we did not conduct the follow-up examination in 2023 because the patient could not tolerate the long car rides. Therefore, we phoned his family and were told the activity of daily living (ADL) in this patient was still well, and no warning symptoms of colon cancer had recurred until now.

## Discussion

To our knowledge, this is the first report of the successful treatment of colon cancer in an older man using multifrequency endoscopic minimally invasive therapy. Older patients diagnosed with advanced CRC are less likely to receive standard antitumour therapies, such as cytotoxic chemotherapy and biological therapy, and the proportion of patients receiving treatment declines with advanced age (5, 6). In this case, we originally aimed to prevent colonic obstruction by the tumour and to maintain the basic quality of life of the patient through endoscopic palliative care. However, the final successful treatment of cancer by multifrequency endoscopic resection provided an easily acceptable and low-risk protocol for the treatment of CRC in older patients.

Relevant literature was reviewed to explore potential reasons. Biller et al. reported that the 5-year survival rate of patients with metastatic CRC is less than 20% (7). The colon cancer in this patient was localised in the lumen when diagnosed and did not metastasise to the lymph nodes or distant sites. The recognition of warning symptoms and accurate diagnosis of colorectal cancer are important, and there were no signs of metastasis during the 4-year follow-up treatment and re-examination, providing an opportunity for long-term endoscopic invasive therapy. Endoscopic invasive therapy is less harmful and safer for older patients than traditional surgical care because of postoperative pain and the risk of complications such as wound infection and poor healing caused by the surgery (8). Older patients also are more liable to postoperative surgical site infections and poor recovery after colorectal surgery than young patients because of their poor nutritional absorption and weak resistance to pathogens (9–11). And the complications are reported to be associated with many adverse outcomes like increasing patient costs and length of hospital stay, promoting the incidence of sepsis, or even causing death (12, 13). Recently, minimally invasive endoscopic therapy has been considered for gastrointestinal tumours treatment (14–18). APC has been reported to be effective in treating superficial oesophageal squamous cell carcinoma in patients with severe concomitant disease (15). Endoscopic submucosal dissection (ESD) and endoscopic full-thickness resection (EFTR) have been found as available ways for early colorectal cancer confined to the mucosa or submucosa (16–18). The appropriate frequency of endoscopic therapy for this patient may also be one of the reasons for successful treatment. Moreover, the tumour location of this patient was in the descending colon, and Zhang et al. reported that the prognosis of patients with left-sided colon cancers was better than that of patients with right-sided colon cancers, regardless of stage (19), which may also have contributed to the successful treatment of the patient. Therefore, the primary location of the colon cancer, its staging, the accurate diagnosis and the appropriate choice of therapy were combined to account for the favourable outcome in this patient.

In conclusion, this case implies that invasive endoscopic therapy may be feasible to treat colon cancer in older populations. However, there are also limitations, such as how frequently and to what extent endoscopic resection should be performed and which older patients would benefit. Moreover, the early and accurate diagnosis of colon cancer is also vital to provide the chance for minimally invasive therapy, and some studies have reported deep learning algorithms have the potential to improve the accuracy and efficacy of CRC detection (20, 21). Therefore, further clinical practice and investigations are needed to apply deep learning algorithms to the classification and diagnosis of CRC and invasive endoscopic therapy for the therapy of CRC.

## Data availability statement

The original contributions presented in the study are included in the article/**Supplementary Material**. Further inquiries can be directed to the corresponding author.

## Ethics statement

The studies involving humans were approved by The Ethical Committee of the 909th hospital. The studies were conducted in accordance with the local legislation and institutional requirements. The participants provided their written informed consent to participate in this study. Written informed consent was obtained from the individual(s) for the publication of any potentially identifiable images or data included in this article.

## Author contributions

NZ: Conceptualization, Methodology, Software, Writing – original draft. LZ: Conceptualization, Methodology, Writing – original draft. YL: Conceptualization, Methodology, Writing – review & editing. XC: Writing – review & editing. BZ: Writing – review & editing. CW: Supervision, Writing – review & editing. HZ: Writing – review & editing. QT: Writing – review & editing. MZ: Methodology, Writing – review & editing.
