# Supplementary material for: Case report: Successful treatment of advanced colon cancer in an eighty-year-old man with long-term and multi-stage endoscopic minimally invasive therapy
Source: Front Oncol. 2024 Feb 20;14:1367173. doi: 10.3389/fonc.2024.1367173 (PMC10914250; doi:10.3389/fonc.2024.1367173)
Supplement: Supplementary file 1 [file Image_1.pdf]

Supplementary Material

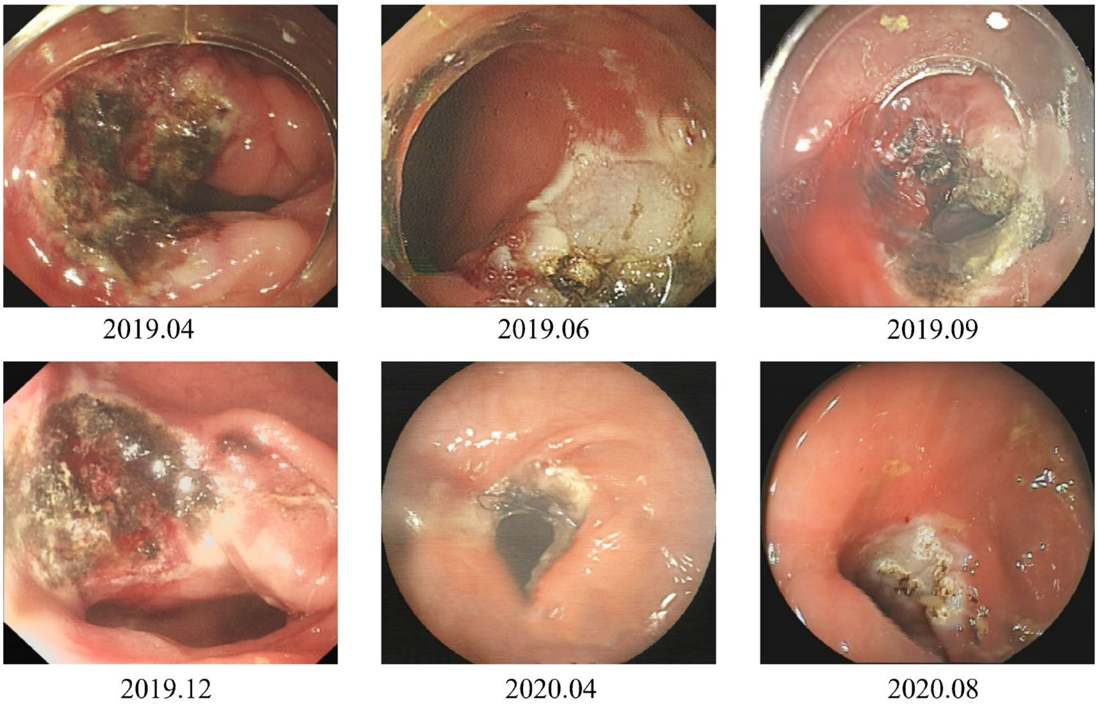

Supplementary Figure1. Long-term and multi-stage endoscopic minimally invasive therapy.
